# Clinical Profile, Functional Outcome, and Mortality of Guillain-Barre Syndrome: A Five-Year Tertiary Care Experience from Nepal

**DOI:** 10.1155/2019/3867946

**Published:** 2019-06-02

**Authors:** Saroj Kumar Bhagat, Shrey Sidhant, Mukesh Bhatta, Ashish Ghimire, Bhupendra Shah

**Affiliations:** ^1^B.P. Koirala Institute of Health Sciences, Nepal; ^2^Department of Pediatrics and Adolescent Medicine, B.P. Koirala Institute of Health Sciences, Nepal; ^3^Department of Anaesthesiology and Critical Care, B.P. Koirala Institute of Health Sciences, Nepal; ^4^Department of Internal Medicine, B.P. Koirala Institute of Health Sciences, Nepal

## Abstract

**Introduction:**

Guillain-Barre syndrome is the most common cause of acute flaccid paralysis in the adult population. It occurs at the rate of 0.34 to 4 per 100000 individuals. This study was conducted to determine the clinicoepidemiological profile and outcome of the patients with Guillain-Barre syndrome.

**Materials and Methods:**

We conducted a retrospective study of patients with Guillain-Barre syndrome, presented at B.P. Koirala Institute of Health Sciences, a tertiary care centre in eastern Nepal, from January 2013 to December 2017. All patients diagnosed with Guillain-Barre syndrome were included in this study. The handwritten case record files of the study population were retrieved from medical record section of the institute.

**Results:**

Of 31 patients with Guillain-Barre syndrome, the mean age of patients was 17±12 years. The most common presenting symptom of study population was ascending paralysis (93.5%). Respiratory failure requiring mechanical ventilation occurred in 16.1%. The common variants are AIDP and AMAN. Respiratory tract infection (29%) was the most common antecedent event. The in-hospital mortality of Guillain-Barre syndrome was 6.45%.

**Conclusion:**

Guillain-Barre syndrome is commonly seen in the young population. The most common symptom of Guillain-Barre syndrome was ascending paralysis. The in-hospital mortality rate of patients with GBS was 6.45%.

## 1. Introduction

Guillain-Barre syndrome (GBS) is the leading cause of acute neuromuscular weakness in the developed world [1]. The mortality rate of GBS was 5-15%. However, the data regarding clinicoepidemiological profile and outcome of the patients with GBS from developing countries are limited. Identifying patients with GBS having poor prognostic factor aids in proper utilization of the limited resources while managing the patients with GBS. To fulfill this unmet need in the management of patients with GBS in resource-limited countries, we have conducted a retrospective study among patients with GBS admitted to the Department of Internal Medicine, Department of Anesthesiology and Critical Care, and Department of Pediatrics and Adolescent Medicine at B.P. Koirala Institute of Health Sciences, Dharan, Nepal, to determine the clinicoepidemiological profile and outcome of patients with GBS.

## 2. Materials and Methods

### 2.1. Study Design

We conducted this retrospective study at B.P. Koirala Institute of Health Sciences, a pioneer tertiary care centre in eastern Nepal. We enrolled patients with Guillain-Barre syndrome diagnosed by treating physician and admitted to Department of Internal Medicine, Department of Pediatrics and Adolescent Medicine, and Intensive Care Unit for the period of five years from 1st January 2013 to 31st December 2017. Patients with previous trauma leading to paresis, previous neuromuscular weakness, poliomyelitis, periodic paralysis, transverse myelitis, and diphtheria and porphyria renal tubular acidosis were excluded from the study.

### 2.2. Study Procedure

We retrieved handwritten case record files from the record section after getting permission from the head of Department of Internal Medicine, Pediatrics, Intensive Care Unit and hospital director of the institute. We recorded the data regarding the epidemiology, clinical profile, baseline vital parameters, laboratory values, electrodiagnostic finding, treatment received, and outcome of the patients with GBS. Diagnosis of Guillain-Barre syndrome was assessed by Brighton criteria and classified into different levels of certainty ranging from level 1 to level 4 as shown in Table 1. History regarding the triggering events were taken from the handwritten record files. Baseline laboratory investigation reports of the study population were noted. The severity of disease was assessed by Medical Research Council sum score at admission which includes power assessment of the deltoid, biceps, wrist extensor, iliopsoas, quadriceps, and tibialis anterior with maximum score of 60. The pattern of nerve conduction velocity was noted as demyelinating, axonal, or combined.

### 2.3. Outcome Measures

The primary outcome of the study was the proportion of patients who had in-hospital mortality, and the secondary outcome was a clinicoepidemiological profile and functional outcome of patients with Guillain-Barre syndrome. Functional outcome of the patients was assessed by Hughes motor scale at the time of discharge. Hughes motor scale ranges from 0 to 6 where o is asymptomatic, 1 is having mild signs or symptoms but able to run, 2 is able to walk unaided for 5 meters, 3 is able to walk 5 meters with support, 4 is bedridden or chairbound, 5 is requiring ventilator assistance, and 6 is death of the patient.

### 2.4. Statistical Analysis

Data from the proforma were filled into MS Excel 2010 and analyzed by SPSS 20 version. For descriptive analysis frequency, percentage, mean, median, standard deviation, and interquartile range were calculated and presented in tabular form whereas for inferential statistics independent t-test was applied as per need to find out the difference between groups. We consider values as statistically significant at a 95% confidence interval if P<0.05.

## 3. Result

### 3.1. Level of Certainty of Diagnosis

**Figure 1:**
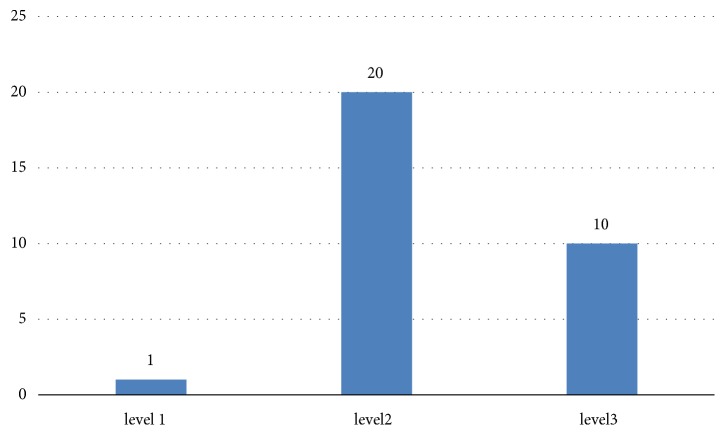
Brighton criteria level of diagnostic certainty of diagnosis of Guillain-Barre syndrome (n-31).

As shown in Figure 1, of 31 patients with GBS, the majority of the patients had Brighton criteria level 2 certainty of diagnosis (64.5%). 32% of study population had level 3 diagnosis certainty.

### 3.2. Clinical Symptoms

As shown in Table 2, in this retrospective study, among patients with GBS, the majority of the patients were female (51.6%). The most common presenting symptom was ascending paralysis that occurred in 29 patients (93.5%). The other common symptoms presented were sensory symptoms (22.6%), respiratory failure (16.1%), and dysphagia (12.9%).

### 3.3. Triggering Events

As illustrated in Table 2, the most common antecedent event in this study was respiratory tract infection (29%) followed by surgery (9.7%). Diarrhea as an antecedent event was reported only in 1/31 patient (3.2%). However, the antecedent event among 48.4% was unknown.

### 3.4. Severity of Involvement

The common complications noted among the study population were respiratory failure (16.1%) and autonomic dysfunction (12.9%). The mean Medical Research Council sum score which assesses the motor power was 38 (SD-11) as illustrated in Table 2.

### 3.5. Nerve Conduction Study Finding

As shown in Table 2 the common GBS variants according to nerve conduction studies were AIDP (19.4%) and AMSAN (19.4%). However the nerve conduction studies of 61.2% of the study population were not known.

### 3.6. Outcome of the Study Population

**Figure 2:**
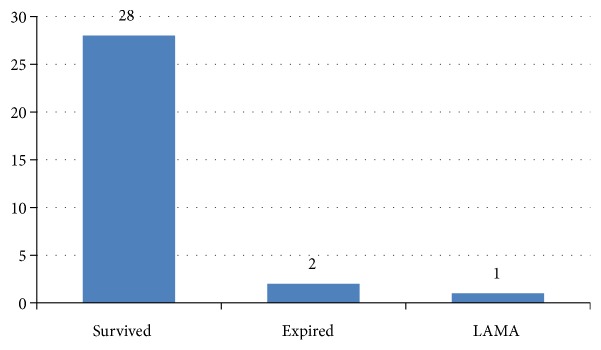
Outcome of patients with Guillain-Barre syndrome (n: 31).* Abbreviation*. LAMA: leave against medical advice.

**Figure 3:**
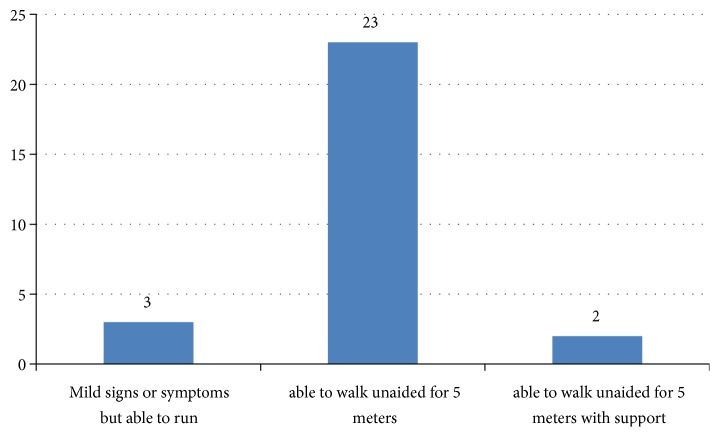
Hughes functional outcome of patients with Guillain-Barre syndrome (n: 28).

As shown in Figure 2, among 31 patients with GBS, 90 percentage (28/31) of the patients survived. The in-hospital mortality rate of patients with GBS in this study was 6.45% (2/31). As shown in Figure 3, among the patients with GBS who survived, the majority of patients were able to walk unaided for 5 meters (26/28). As elucidated in Table 3, the patients with unfavourable outcome presented earlier than favourable outcome group; however, the difference is not statistically significant (160 hours* vs.* 315 hours, P value: 0.355). The MRC sum score of favourable outcome patients was 38 and that of unfavourable outcome patients was 44. Similarly, the length of stay of favourable outcome group is more than that of unfavourable outcome group, which is not statistically significant (11 days* vs.* 2 days, P value: 0.431).

## 4. Discussion

In our study, among 31 patients with GBS the mean age of patients was 17 years (SD-12). The common symptoms were ascending paralysis in 29 patients (93.5%), sensory disturbance in 7 patients (22.6%), and respiratory failure in 5 patients (16.1%). The most common antecedent event was respiratory tract infection (29%) followed by surgery (9.7%). The in-hospital mortality of patients with GBS was 6.45%. The majority of patients with GBS were able to walk unaided before discharge from hospital (92.85%).

In our study, the mean age of patients with GBS was 17 years which was lower than that reported by Dhungana K et al. (35 years) in their prospective study done in central Nepal [2] and Kabir ATMH et al. (30 years) from Bangladesh [3]. This contrast in the findings is because of the recruitment of pediatric age group in our study which was excluded in their study. Our data corroborate with the study of Kalita J et al. (25 years) in Sanjay Gandhi Post Graduate Institute of Medical Science in Lucknow, India [4]. Our study showed that GBS can occur in the young population.

The most common symptom of GBS was ascending paralysis (93.5%). The other symptoms noted were sensory symptoms (22.6%), respiratory failure (16.1%), and dysphagia (12.9%). The prevalence of ascending paralysis in our study was similar to the data of Mateen J F et al. from India [5] (100%) and Xiaowen Li et al. (83.33%) from China [6]. Percentage of patients who needed mechanical ventilation due to respiratory failure was similar (13.9%) to that found by Zhang B et al. from China [7]. However, it is less (55.9%) than data of study conducted by Yakoob MY et al. at a tertiary care centre in Pakistan [8]. The increased prevalence of respiratory failure among patients enrolled by Yakoob MY et al. was due to hospital-acquired pneumonia. Respiratory failure is one of the deadliest complications of GBS caused by weakness of pharyngeal and diaphragmatic muscle, pneumonia, and autonomic dysfunctions.

The in-hospital mortality of GBS in our study was 6.45%. Our data corroborate with the findings of studies conducted by Sharma KS et al. (7.4%) in Nepal among the pediatric population [9] and Kalita J et al. (6.8%) at Sanjay Gandhi Post Graduate Institute of Medical Sciences, Lucknow, India [4]. The mortality rate in our study was significantly higher than that reported by Alshekhlee A et al. (2.58%) in their study among the US population [10]. The discordance in the mortality rate reflects the need for resources in developing countries. In our study the majority of patients had a good functional outcome (92.8%), which was similar to the findings of Rees JH et al. (88%) from South East England [11]. The mean age of patients with GBS having favourable outcome is less than that of unfavourable outcome group (16* vs.* 23, p: 0.355); however, the difference was statistically insignificant. This was similar to the finding by Zhang B et al. which showed the poor prognosis among patients with GBS of advancing age (69* vs.* 39, p-0.008) [7]. Further studies are required to determine the age as one of the prognostic features of patients with GBS. From our study no predictors are statistically significant between a favourable outcome and unfavourable outcome group; this might be due to the small sample size in our study.

*Strength*. It is among the few studies in the country to deliver data regarding the clinical-epidemiological profile and outcome of the GBS.

*Limitations*. This was a retrospective study; long-term prognosis of GBS could not be assessed. The majority of the patients did not undergo electrophysiological test; thus we could not identify variant of GBS. NCV finding available in the files was not sufficient to categorize the variant of GBS perfectly. We could not do the work-up for infectious etiology of the GBS as the serological studies were not available in the institute. The majority of the patients did not undergo MRI of the brain which might have ruled out bilateral anterior cerebellar artery stroke. The sample size is small. The cause of death of the patients could not be assessed.

*Future Direction*. We recommend a prospective study among patients with GBS to determine their long-term prognosis. Interventional studies to assess the effectiveness of therapeutics in GBS is a need of future studies.

## 5. Conclusion

GBS was seen in all age groups with slight female predominance. The majority of the patients had an antecedent history of respiratory tract infection and surgery. The common symptoms were ascending paralysis, sensory symptoms, and dysphagia. The in-hospital mortality rate of patients with GBS was 6.45%. The majority of the patients with GBS had a good functional outcome.

## Figures and Tables

**Table 1:** Brighton criteria level of diagnostic certainty of Guillain-Barre syndrome.

Variables	Level 1	Level 2	Level 3	Level 4
Bilateral and flaccid weakness of limbs	+	+	+	+/-
Decreased or absent deep tendon reflex in weak limbs	+	+	+	+/-
Monophasic course and time between onset-nadir 12 hours and 28 days	+	+	+	+/-
CSF cell count < 50/microliter	+	+	-	+/-
CSF protein concentration> normal value	+	+/-	-	+/-
NCS finding consistent with one of the subtypes of GBS	+	+/-	-	+/-
Absence of alternatives diagnosis for weakness	+	+	+	+

Abbreviations. CSF: cerebrospinal fluid, NCS: nerve conduction study, GBS: Guillain-Barre syndrome.

+: present, -: absent.

**Table 2:** Clinicoepidemiological study of Guillain-Barre syndrome (n: 31).

Variables	Value	Percentage
Gender		
Male	15	48.4
Female	16	51.6

Clinical profile		
Ascending paralysis	29	93.5
Sensory disturbance	7	22.6
Respiratory failure	5	16.1
Dysphagia	4	12.9
Autonomic dysfunction	4	12.9
Bladder involvement	4	12.9
Cranial nerve involvement	3	9.7

Antecedent Event		
Respiratory tract infection	9	29
Surgery	3	9.7
Recent vaccination	2	6.5
Diarrhea	1	3.2
Urinary tract infection	1	3.2
Unidentified	15	48.4

NCV		
AIDP	6	19.4
AMSAN	6	19.4
Not done	19	61.2

Abbreviations. NCV: nerve conduction velocity, AIDP: acute inflammatory demyelinating polyneuropathy, AMSAN: acute motor sensory axonal neuropathy.

**Table 3:** Baseline characteristics of patients with Guillain-Barre syndrome (n: 31).

Variables	Total participants (n-31)	Favourable outcome (n-28)	Unfavourable outcome ^#^(n-3)	P value
	Mean ± SD	Mean ± SD	Mean ± SD	
Age	17 ± 11	16 ± 10	23 ± 19	0.355
Time	300 ± 498	315 ± 521	160 ± 154	0.616
MRC score	38 ± 11	38 ± 11	44 ± 15	0.342
SBP	113 ± 16	114 ± 16	110 ± 10	0.655
DBP	74 ± 12	74 ± 13	73 ± 6	0.933
Temperature	98 ± 0.7	98.2 ± 0.8	97.9 ± 0.1	0.556
Pulse rate	96 ± 17	97 ± 18	90 ± 8	0.539
SpO2	95 ± 3	96 ± 4	95 ± 2	0.771
Length of hospital stay	10 ± 19	11 ± 21	2 ± 1	0.431

Abbreviations. MRC: Medical Research Council, SBP: systolic blood pressure, DBP: diastolic blood pressure, SpO2: oxygen saturation, SD: standard deviation. #: including patients who were expired or went on leave against medical advice.

## Data Availability

The data used to support the findings of this study are available from the corresponding author upon request.
